# Novel non intrusive continuous use ZeBox technology to trap and kill airborne microbes

**DOI:** 10.1038/s41598-021-02184-4

**Published:** 2021-11-23

**Authors:** Kruttika S. Phadke, Deepak G. Madival, Janani Venkataraman, Debosmita Kundu, K. S. Ramanujan, Nisha Holla, Jaywant Arakeri, Gaurav Tomar, Santanu Datta, Arindam Ghatak

**Affiliations:** 1 Biomoneta Research Private Limited, Bangalore, 560065 India; 2 Department of Veterinary Microbiology and Preventive Medicine, Iowa State University, Ames, IA 50011 USA; 3 Mechanical Engineering Department, Indian Institute of Science, Bangalore, 560012 India; 4 Bugworks Research, Bangalore, 560066 India

**Keywords:** Epidemiology, Microbiology

## Abstract

Preventing nosocomial infection is a major unmet need of our times. Existing air decontamination technologies suffer from demerits such as toxicity of exposure, species specificity, noxious gas emission, environment-dependent performance and high power consumption. Here, we present a novel technology called “ZeBox” that transcends the conventional limitations and achieves high microbicidal efficiency. In ZeBox, a non-ionizing electric field extracts naturally charged microbes from flowing air and deposits them on engineered microbicidal surfaces. The surface’s three dimensional topography traps the microbes long enough for them to be inactivated. The electric field and chemical surfaces synergistically achieve rapid inactivation of a broad spectrum of microbes. ZeBox achieved near complete kill of airborne microbes in challenge tests (5–9 log reduction) and $>90\%$ efficiency in a fully functional stem cell research facility in the presence of humans. Thus, ZeBox fulfills the dire need for a real-time, continuous, safe, trap-and-kill air decontamination technology.

## Introduction

Microbial load (bacteria, viruses, spores and fungi) in our living, working and hospital space must be reduced to mitigate the transmission of airborne infections. As per CDC (Center for Disease Control, USA)’s recommendation (https://www.cdc.gov/niosh/topics/hierarchy/default.html), eliminating microbes at the source as and when produced is the first line of defense against spread of infections. Filtration, electrostatic precipitation, bactericidal gas spraying, ultra-violet germicidal irradiation (UVGI, employing $\sim 254$ nm radiation), plasma discharge and photo-catalytic oxidation (PCO) are the currently available air decontamination technologies^1^. While some are microbicidal, others only trap microbes. Filtration^2^ and electrostatic precipitation^3^ belong to the latter category. Microbes trapped inside filters can multiply in situ^4–8^; such filters are detrimental to indoor air quality and hazardous during their disposal. They also offer high flow resistance which translates to high operating power consumption^9,10^. Electrostatic precipitation uses electric field to attract and trap aerosols pre-charged by corona discharge, but which produces noxious gases like ozone^3,11^. Its microbicidal action is dubious; in fact electrostatic bioaerosol samplers capture microbes that remain viable^12–14^. However, because of its low flow resistance, it consumes less power per unit of clean air delivered compared to filtration^3^. Filters made of anti-bacterial fibers have also been developed^15–20^ but their performance remains to be proven under realistic indoor conditions.

Bactericidal gas spraying, UVGI, plasma discharge and PCO are microbicidal technologies. Although bactericidal gases and UVGI can sterilize an entire room, they cannot be deployed in human presence. UVGI is used to sterilize upper room air and air circulating through ventilation ducts. However, microbicidal action of UVGI depends on environmental parameters such as humidity^21–23^, is species-specific^24^ and requires a minimum duration of exposure to microbes^25^. Exposure of humans to UVGI (due to faulty design, deployment or use of UVGI devices) can damage their eyes and skin^26–29^. UVGI is used to kill microbes trapped on a filter’s surface^30,31^ but then it cannot reach microbes residing beneath the surface. Plasma discharge^32^ and PCO^34,35^ both generate ions and/or reactive species, respectively using gas discharge and reaction with an irradiated catalyst. However, they also generate NO$_X$ and ozone^1^ and additional methods are necessary to mitigate them^33^. In PCO, convection of gas to the catalyst and the subsequent adsorption, reaction and release of reactive species into the bulk flow is the bottleneck process^36^, which results in low clean air delivery rates^1^.

Given the importance of eliminating airborne infection, a technology that is safe, suitable for continuous use and efficient against a wide variety of airborne microbes is desirable. Here, we describe such a novel technology called “ZeBox”; the name derives from the **Ze**ta-potential possessed by microbes, which property is pivotal in trapping them inside the **Box**-shaped device. In the following, we discuss the working mechanism of ZeBox and demonstrate its efficacy in chamber tests and field studies against a variety of microbes.

## Results

### Electrode plates with engineered chemical surfaces form the kill cassette

**Figure 1:**
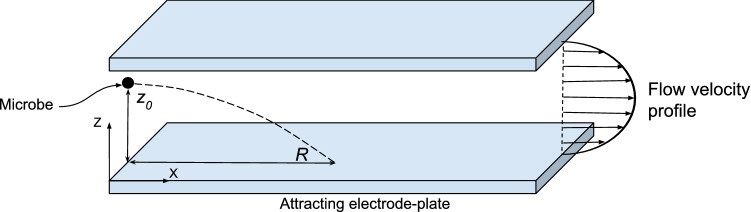
Microbe motion in electric field. A charged microbe deviates from the flow direction due to the electric field between electrode-plates. (created using Google Draw, https://docs.google.com).

A row of flat plate electrodes (10.9 cm $\times$ 30 cm) with alternating polarity are assembled inside a cuboid shaped box with open ends for transmitting flow. A three dimensional hydrocellular microbicidal composite material (US patent no. US 9566363B2, licensed) is layered on to the electrodes. A non-ionizing 3 kV/cm electric field is set up between electrodes by applying direct-current voltage between them. Microbes are trapped and killed inside this “kill cassette”. Axial fans pull microbe laden ambient air through the kill cassette and between the electrode-plates, as shown schematically in Fig. 1.

### Electric field extracts charged microbes from the flow

Microbes are naturally charged^37,38^; therefore, in an electric field, they are impelled towards the electrode of opposite polarity. Figure 1 depicts this process schematically. Here, *X*-axis points along the flow and *Z*-axis points away from the attracting electrode. A microbe initially at distance $z_0$ from the attracting electrode travels a distance *R* in the streamwise direction, called its “range”, as it descends to $z=0$. Whether or not the microbe hits the electrode depends on its length, the microbe’s initial distance $z_0$, strength of the electric field, charge on the microbe and the type of flow (laminar or turbulent). The Reynolds number for the flow between electrodes in ZeBox is $\sim 10^3$ and a rectangular duct flow (or even plane Poiseuille flow) undergoes transition at this Reynolds number and could be turbulent^39,40^. Analyzing microbe’s motion in a turbulent flow is difficult because of its complicated, stochastic nature. Supplementary information S1 analyzes microbe’s motion and its maximum range in a laminar flow instead. The settling speed is obtained by equating electrostatic and drag forces on the microbe, while also accounting for its changing streamwise speed as it settles (the steady laminar velocity profile of the background flow being known); the result of the analysis is a universal dimensionless curve for microbe’s range, refer Supplementary Fig. 1, from which the efficiency of ZeBox may also be computed given its operating parameters.

**Figure 2:**
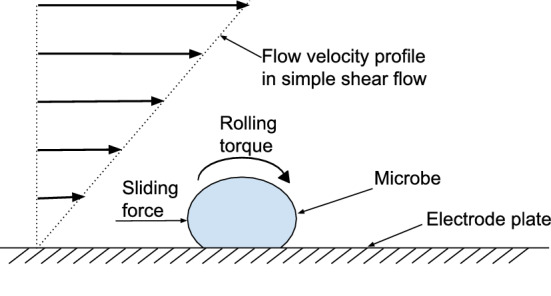
Microbe slippage on solid surface. Microbes can slide and roll over a flat surface due to hydrodynamic force and torque exerted by the flow. (created using Google Draw, https://docs.google.com).

Earlier studies on resuspension of dust from flat surfaces due to a flow show that, whenever the hydrodynamic force and torque exerted by the flow exceed those that keep the particles attached to the surface (for example, Van der Waals force), the particles can either detach by lifting off or slide and roll on the surface^41,42^. In our case, lifting off of microbes from the electrode is unlikely due to the strong electric field, but they can nevertheless slide and roll and thus escape away due to the electrode’s finite length (refer Fig. 2). Since the microbicidal surface requires a minimum duration of contact to inactivate microbes depending on how sensitive or hardy it is, a fraction of the deposited microbes could escape while still viable. Therefore, the ability of the microbicidal surface to trap and hold microbes until they are inactivated becomes important.

### Three dimensional topography of the microbicidal surface traps the microbe

**Figure 3:**
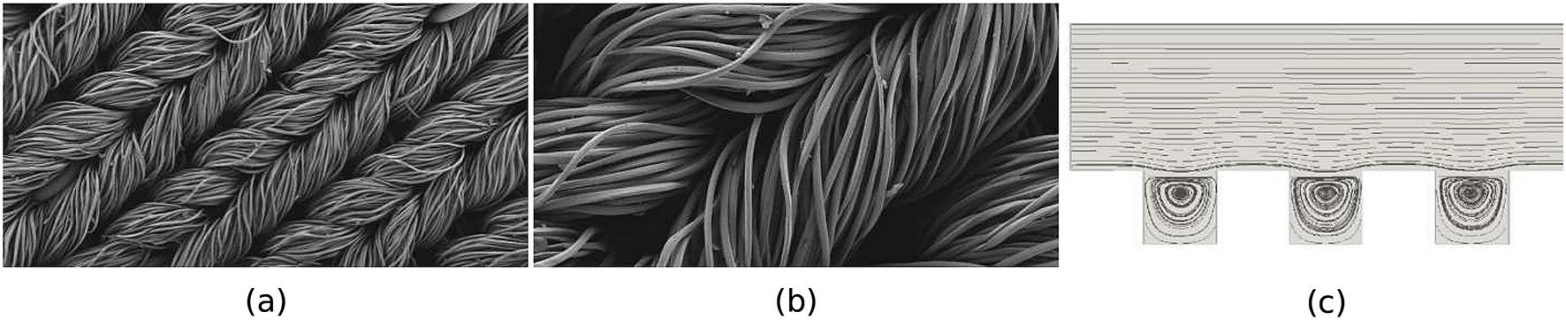
Uneven topography of microbicidal fabric. (**a**) SEM photograph at $118 \times$ magnification. (**b**) SEM photograph at $363 \times$ magnification. (**c**) Recirculating flow due to uneven topography simulated in OpenFOAM-7. (**a**, **b** are outputs of the Field Emission Scanning Electron Microscope (MERLIN Compact VP from M/s.Carl Ziess), **c** created using Paraview (https://www.kitware.com/platforms/#paraview)).

The microbicidal surface employed in ZeBox has a highly uneven topography at the microbial scale, populated with well-like depressions to trap and hold microbes. Figure 3a, b show the scanning electron microscope (SEM) images of the surface at different magnifications appropriate to the microbial scale. Figure 3c shows streamlines in a numerically simulated two dimensional flow (using OpenFOAM-7) over a surface with square shaped wells, to qualitatively illustrate the kind of flow obtained over an uneven topography. Any bulk flow may be approximated as simple shear flow sufficiently close to a solid surface. A simple shear flow is characterized entirely by its shear rate, estimated as *U*/*H* for our case; $U\approx 1$ cm/s is the average flow speed between electrode plates and $H=1$ cm is the gap between them. The flow Reynolds number based on shear rate and characteristic dimension of the square well, *d*, is $Re\equiv (U/H)d^2/\nu$, where $\nu =1.5\times 10^{-5}$ m$^2$/s is the kinematic viscosity of air. From Fig. 3b, $d\sim 10~\mu$m, which yields $Re\sim 10^{-5}$. A simple shear flow was imposed on the flow domain (refer Fig. 3c) by moving its uppermost boundary horizontally at constant speed to achieve the aforementioned Reynolds number. The important feature of the flow for our purpose is the recirculating region set up within the wells, in which the streamlines of the flow form closed loops. This feature is quite general for a flow over an uneven topography and which presumably enhances the efficacy of the microbicidal surface further in regard to trapping microbes. Once the microbe falls into one of the wells, brought there either in the course of its rolling over the surface or directly by the electric field, the recirculating flow can confine it to the well for a sufficiently long duration.

Table 1 shows the efficacy of microbicidal surfaces (in terms of $\log _{10}$ reduction, where *n*-$\log _{10}$ reduction implies reduction in the initial microbial load by a factor of $10^n$) with different topographies, which we call 2-D and 3-D surfaces, in flow experiments. A 2-D surface is a single layer of cotton fabric while a 3-D surface is a multilayered 90:10 polyethylene : cotton fabric. In the presence of electric field, 3-D microbicidal surface performs better than the 2-D surface. When the electric field is absent, the microbes are not extracted from the flow and hence both surfaces perform similarly.

**Table 1:** Effect of electric field and fabric topography in flow experiments

	Microbial load reduction ($\log _{10}$ scale)	
	3-D surface	2-D surface
Without electric field	2.82 $^{\pm 0.74}$	2.13 $^{\pm 0.2}$
With electric field	9.42 $^{\pm 1.02}$	4.68 $^{\pm 0.88}$

Log$_{10}$-reduction in viable microbial load (*E. coli*) achieved by ZeBox with 3-D and 2-D microbicidal surfaces in 10 min. Applied electric field = 3 kV/cm. Superscripts show standard deviation. Initial load was $10^8$–$10^{10}$ microbes.

**Table 2:** Effect of electric field in spot experiments

Time (mins)	Microbial load reduction ($\log _{10}$ scale)	
	With electric field	Without electric field
2	3.00$^{\pm 0.39}$	0.87$^{\pm 0.44}$
5	5.71$^{\pm 0.19}$	1.86$^{\pm 0.78}$
10	8.83$^{\pm 0.69}$	2.56$^{\pm 1.17}$

Effect of 3 kV/cm electric field on the $\log _{10}$-reduction in viable microbial load (*E. coli*) over the microbicidal surface in spot experiments. Superscripts show standard deviation. Initial load was $10^8$–$10^{10}$ microbes

**Figure 4:**
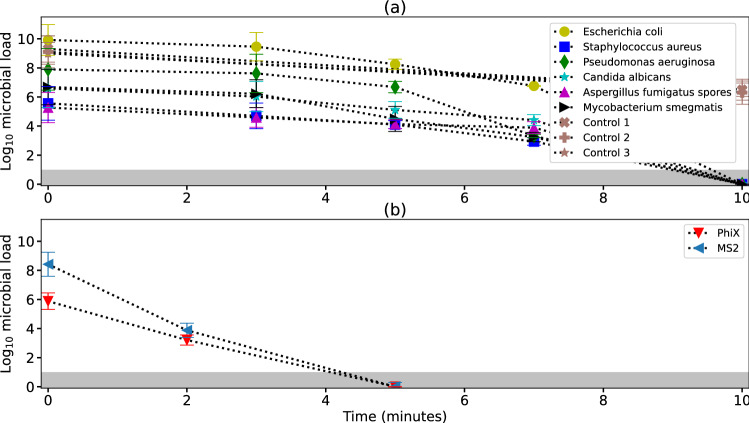
Reduction in microbial load inside test chamber. (**a**) Reduction in microbial load except viruses. The shaded region indicates limit of detection (LoD). Control 1, 2, 3 refer to control experiments employing respectively microbicidal surface without electric field, control surface with electric field and control surface without electric field. (**b**) Reduction in viral load. (created using Matplotlib module in python language^55^).

### Electric field and chemical microbicidal-surfaces synergistically achieve rapid inactivation of microbes

In contrast to electrostatic precipitators, the applied electric field in ZeBox plays two roles: it pulls microbes from the flow on to the microbicidal surface and then accelerates their subsequent inactivation. Table 2 shows $\log _{10}$-reduction in the microbial load in spot experiments, with 3 kV/cm electric field applied between electrodes. The microbicidal surface achieves the highest reduction in microbial load in the presence of the electric field. Quaternary ammonium compounds (QAC) are membrane-active agents which inactivate microbes by targeting their cytoplasmic membrane^43–46^, but first, they must breach the outer cell wall. In the present design, QAC is tethered to the 3-D surface by long flexible chains, which presumably helps the QAC to orient itself to puncture holes in the microbe. The external electric field increases the trans-membrane voltage of the cell above its resting value, leading to an electric current that presumably flows through these pores as they form the path of least resistance. This current flow may be analogous to the electroporation of bacteria in which the pores formed in the cell wall are stabilized^47^. The intracellular components then leak from the pores, as is seen in the SEM pictures. This process leads to the irreversible killing of the cells. Therefore, the chemical surface in tandem with the electric field displays an enhanced electro-chemical microbicidal action compared to what they would have achieved separately.

### ZeBox rapidly reduces microbial load in chamber tests

The capability of ZeBox to decontaminate a closed space containing airborne microbes was determined by challenge tests^48^. A broad spectrum of microorganisms was employed in the test – standard gram-positive and gram-negative bacteria of ESKAPE group (*Escherichia coli*, *Staphylococcus aureus*, *Pseudomonas aeruginosa*), mycobacterium species (*Mycobacterium smegmatis*), fungal species (*Aspergillus fumigatus* spores and *Candida albicans*) and virus (PhiX 174 coliphage and MS2 coliphage). Among these, MS2 virus is an accepted surrogate for the SARS-CoV2 virus^49,50^. Figure 4 shows the collated data on the variation in $\log _{10}$ microbial load (*n*-$\log _{10}$ microbial load equals $10^n$ microbes) over time after ZeBox was turned on. ZeBox proves to be extremely effective in rapidly decreasing the viable microbial load in a closed space. It achieved 9.9 $\log _{10}$-reduction (i.e. 99.999999999% reduction) of *E. coli* in 10 min (*n* $\log _{10}$-reduction equals reduction by a factor of $10^n$). For other microbes ZeBox brought about 5 to 9 $\log _{10}$-reduction (i.e. 99.999-99.9999999% reduction) of the viable microbial load.

**Figure 5:**
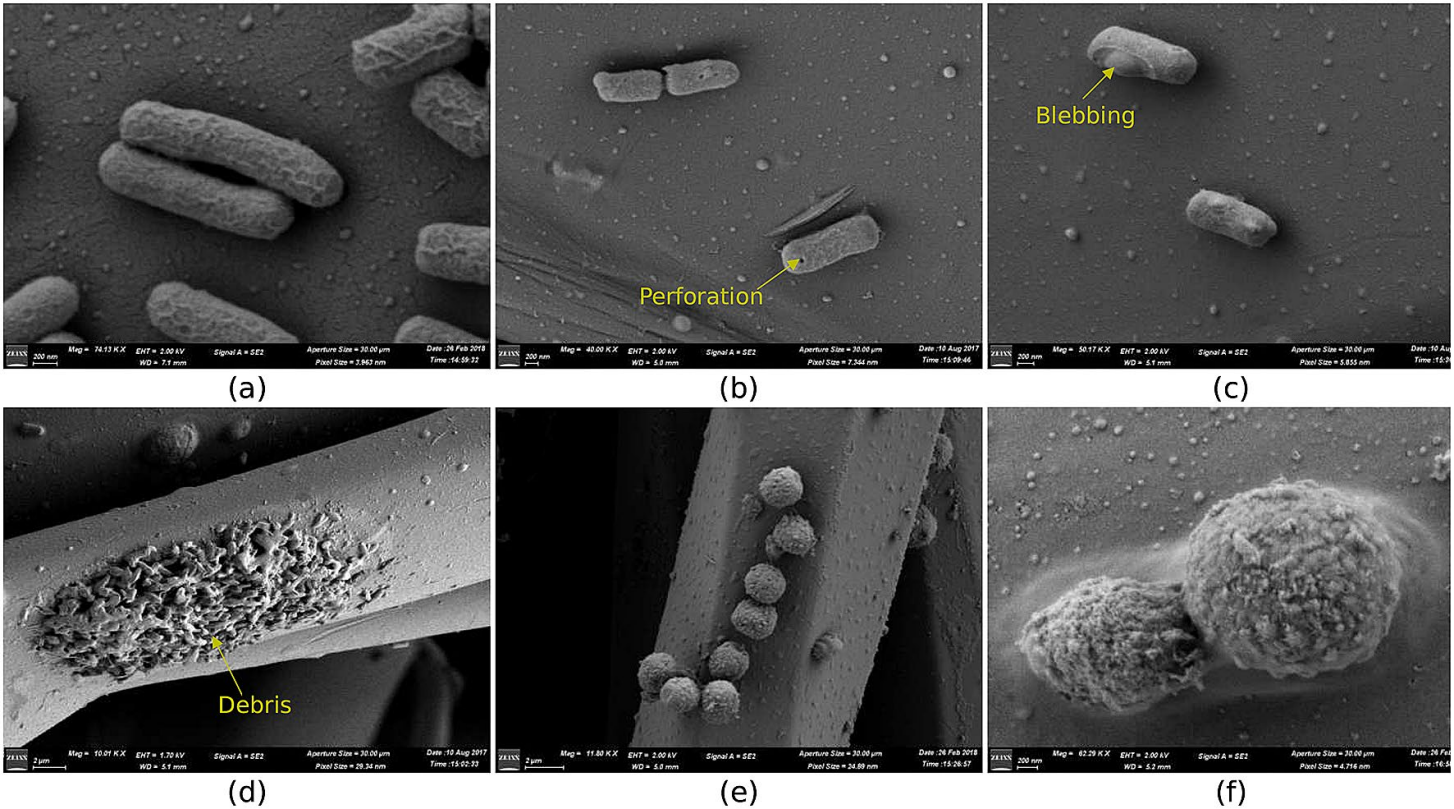
Mechanism of lysing of microbes. (**a**) *E. Coli*, control. (**b**) *E. Coli*, after 5 min, showing perforation. (**c**) *E. Coli*, after 5 min, showing blebbing. (**d**) *E. Coli*, after 10 min, showing accumulated debris. (**e**) *A. Fumigatus*, control. (**f**) *A. Fumigatus*, after 5 min. (output of Field Emission Scanning Electron Microscope (MERLIN Compact VP from M/s.Carl Ziess), annotation inserted using Matplotlib module in python language^55^).

### SEM images of microbicidal action

Scanning electron microscopy (SEM) studies were done to see how microbes trapped on the microbicidal surface are killed. *E. coli* and *A. fumigatus* spores were chosen because they form two extremes on the scale of sensitivity, with spores being hardy. Figure 5a,e show the microbes in control conditions. Due to electro-chemical action at the three dimensional microbicidal surface, their cell membrane undergoes morphological changes followed by complete degradation. Figure 5b,c, obtained after 5 min of contact, reveals puncturing and blebbing of the *E. coli* cell membrane. Ultimately, the cells burst and their intracellular contents spill out (Fig. 5d,f) signaling a complete degradation of the microbes.

**Figure 6:**
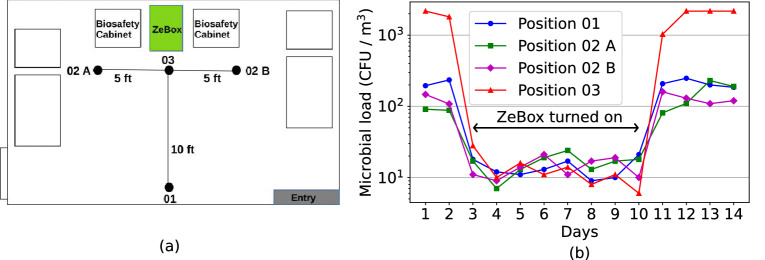
Microbial load reduction in field experiment. (**a**) Setup of the field experiment. Measurement locations are indicated by filled circles in the schematic. (**b**) Reduction in the microbial load. (**a** created using LibreOffice Draw (https://www.libreoffice.org), **b** created using Matplotlib module in python language^55^).

### ZeBox reduces microbial load in open room

ZeBox’s performance was also tested in a real life setting, i.e. in a room with constant influx of microbes from outside or due to internal sources. A working tissue culture laboratory in a building with central air-conditioning, but without High Efficiency Particulate Air (HEPA) filters, was chosen for the purpose. Figure 6a shows the schematic plan-view of the lab and the measurement locations. The working people in the lab were the primary source of microbial contamination. Figure 6b shows that the microbial load at location-03 where tissue culture work was carried out was >3000 CFU/m$^3$ initially. ZeBox reduced the microbial load in the lab to $\sim 10$ CFU/m$^3$ within about 3 h after it was turned on. This low level was consistently maintained so long as ZeBox was operational. When it was turned off at day 10, the microbial load rebounded to its original level. During its operation, ZeBox effectively decontaminated a zone of dimensions $\sim 10$ feet $\times ~ 10$ feet (refer Fig. 6a), which demonstrates its potential to decontaminate a smaller region of interest in a relatively large open room, with uncontrolled movement of personnel and without needing physical partitions.

### ZeBox does not produce ozone

Since ZeBox employs non-ionizing electric field, it does not produce ozone (verified in standardized laboratory tests, data not shown here). This is an immense advantage over conventional microbicidal technologies such as plasma and PCO. Also, it consumes <20 Watt-hour of electric energy during its operation.

## Discussion and conclusions

ZeBox technology exploits the fact that microbes (bacteria, viruses, spores and fungi) are naturally charged and therefore can be readily manipulated by an electric field. Using a non-ionizing electric field, microbicidal surfaces with three dimensional topography and electro-chemical kill mechanism, ZeBox achieves significantly higher microbicidal rate compared to other technologies.

Knowing the total reduction in microbial load, as shown in Fig. 4, is inadequate to gauge ZeBox’s efficacy because any level of decontamination may be achieved given sufficient time. Therefore, an overall microbicidal efficiency must be determined while factoring in the time of operation as well as the volume of the room being decontaminated. Towards this end, we may think in terms of the number of nominal air changes in a room achieved in a given duration and the consequent reduction in microbial load for each air change. In time *t*, *Qt*/*V* number of nominal air changes is achieved, where *Q* is the air flow rate through ZeBox and *V* is the volume of the room. If $\eta$ is the corresponding microbicidal efficiency, then $N_0$ initial number of viable microbes in the room decreases to $N=N_0(1-\eta )^{Qt/V}$ after time *t*. Using this formula and the latest-time data from Fig. 4 whose ordinate is $\log _{10}N$, we may back-calculate $\eta$ for a specified time duration. For experiments with viruses, $Q/V\approx 1.5$ air changes per minute inside the test chamber, which implies 7.5 air changes during the duration of the experiment, refer Fig. 4b. If, for example, we consider PhiX virus then $\log _{10} N=0, \log _{10} N_0\approx 6$ and $Qt/V=7.5$, which gives $\eta = 1-10^{-6/7.5}=84 ~\%$. For the microbes in Fig. 4a, the test chamber was $\sim 5$ times larger (refer “Materials and methods” section), hence the air change rate was lower by the same factor. The microbicidal efficiency of ZeBox lies in the range of 83-99 % for all the tests. Considering the variety of sensitive and hardy microbes employed, ZeBox is about equally effective against all of them. Supplementary information S2 provides a theoretical estimation of the microbicidal efficiency of ZeBox. To estimate the efficiency using the theory provided in Supplementary information S1, the charge on the microbes must be deduced; towards this end, we measured their zeta potential and used the Debye-Hückel theory which governs the distribution of electric potential around a charged particle^54^, in order to relate the microbe’s zeta potential to its charge (refer Supplementary equation (8)). The resulting theoretical estimate of Zebox’s efficiency aligns reasonably well with that deduced from experimental data.

Airborne microbes of size $<2~ \mu$m can remain suspended in air for several hours before settling down and therefore must be inactivated to reduce the transmission of infections. ZeBox technology presents a universal solution because:

- Freely floating microbes are trapped and killed with high efficiency, eliminating the possibility of future growth.
- The airflow is parallel to antimicrobial surfaces with almost no resistance; therefore, unlike HEPA filters, it has low energy utilization.
- There are no chemical emissions or production of free radicals or ozone; the technology is safe for continuous use in the presence of humans and animals.
- It is equally effective for different varieties of sensitive and hardy microbes.

## Materials and methods

### Challenge tests

#### Test setup

An air-sealed test chamber of dimensions 3 ft $\times$ 4 ft $\times$ 3 ft (approximately 1000 liters in volume) was built with multiple sampling and nebulization ports. The environmental parameters such as relative humidity and temperature could be monitored using a probe located inside the chamber. During experiments, various microorganisms were aerosolized using a 6-jet collision nebulizer (MESA LABS, BGI) into the chamber, and the device efficiency was monitored by collecting and measuring microbial concentration at different time intervals. A second test chamber of dimensions 3ft $\times$ 2.5 ft $\times$ 1 ft (approximately 220 liters in volume) placed inside a biosafety cabinet, with similar aerosolization and sampling port configuration, was used for tests with viruses.

#### Cultivation of test microorganisms

To validate the efficiency of the decontamination device, *Escherichia coli* (MTCC 40), *Pseudomonas aeruginosa* (MTCC 424), *Staphylococcus aureus* (MTCC 96), *Candida albicans* (MTCC 584), *Aspergillus fumigatus* (MTCC 2544), *Mycobacterium smegmatis* (MTCC 6), MS2 coliphage (ATCC 15597-B1) and PhiX 174 coliphage (ATCC 13706-B1) were used. For growing *Escherichia coli*, *Pseudomonas aeruginosa* and *Staphylococcus aureus*, Luria broth was used. For growing *Candida albicans*, Potato dextrose broth was used, while for *M. smegmatis*, Middlebrook 7H9 broth was used. For enumeration of *E.coli*, samples were plated on Luria Bertani agar; Cetrimide agar was used as a selective for the growth and isolation of *Pseudomonas aeruginosa*. Cetrimide inhibits the growth of many microorganisms while allowing *Pseudomonas aeruginosa* to develop typical colonies. For quantification of *Staphylococcus*, Mannitol Salt Agar plates were used. *Candida albicans* and *Aspergillus fumigatus* spores were enumerated using Rose-Bengal Chloramphenicol Agar plates. Coliphages were cultivated using standard method described in ATCC manual. For all microbiological nutrient media were manufactured by HiMedia Laboratories, India unless mentioned otherwise.

#### Aerosolization of test microbes

A 6-jet Collison nebulizer (MESA LABS, BGI) was used to aerosolize the test microbes into the test chamber. Dry air from a compressed air cylinder at a pressure of 10 psi was used to operate the nebulizer. The nebulizer produces bioaerosols of a 2–5 $\mu$m diameter that allows them to float in the air present in the test chamber for a definite period. The length of the nebulization period varied depending on the type of experiment and microorganism, but typically ranged between 30–40 min.

#### Sampling of air for viable microbes

The airborne survival of the test microbe and the activity of the air decontamination devices were determined by collecting the air from the chamber at the rate of 12.5 liter/min using SKC biosampler^51^, filled with sterile buffer ($1\times$ Phosphate buffer saline, pH 7.2). Collected samples were analyzed to understand the quantity of viable microorganism present by diluting and plating them onto suitable growth media. The plated samples were incubated at $37\pm 2^{\circ }\hbox {C}$ for bacteria and $25 \pm 2^{\circ }\hbox {C}$ for fungal species and allowed to grow for 18–48 h as mentioned in the ATCC/MTCC manual, individual colonies were enumerated, and the final concentration of the microbial load was calculated thereafter. For enumerating coliphages collected from the chamber, Double agar overlay method was used for subsequent plaque assay^52^. *E. coli* ATCC 15597 and *E. coli* ATCC 13706 were used as a host in plaque assays for MS2 and PhiX174, respectively. Plaques were counted after 24 h incubation at $37 \pm 2^{\circ }\hbox {C}$.

#### Spot experiments

*E. coli* cells were grown in the standard medium. A known titre of cells were spotted onto a 25 mm$^2$ surface and incubated for various time duration, both with and without electric field. Surfaces were resuspended in 500 $\mu$l of sterile 1X PBS, which was then plated on standard agar plates to enumerate the microbes.

#### Limit of detection

Microbial enumeration is guided by two parameters, Limit of Detection (LOD) and Limit of Quantification (LOQ). For the present assays used to quantify the microbial load inside the test chamber, the LOD was 10 CFU for bacterial and fungal load and 5 PFU for viral load. However, LOD is always less than LOQ^53^. In many of our experimental analysis, post operating ZeBox device, the microbial detected numbers were in around LOD and hence, the exact LOQ was often indeterminant.

#### SEM analysis of trapped microbes to decipher the mechanism of kill

3D surfaces were stripped off from the electrode plates post operating the device against E.coli under challenge test under various time course, and treated with 2.5% glutaraldehyde in 0.1 M phosphate buffer (pH 7.2) for 24 h at $4^{\circ }\hbox {C}$. The samples were dehydrated in series of graded ethanol solutions and subjected to critical point drying with CPD unit. The analyzed samples were mounted over the stud with double-sided carbon conductivity tape, and a thin layer of gold coat over the samples was done by using an automated sputter coater (EMITECK K550X Sputter Coater from EM Scientific Services) for 3 min and analyzed under Field Emission Scanning Electron Microscope (MERLIN Compact VP from M/s.Carl Ziess). The set parameters were: Working Distance = 5–6 mm, EHT range = 2–4 kV, Range of Magnification = 70 KX, detectors = SE2 And InLens, machine under high vacuum.

### Field tests

#### Air sample collection

A working tissue culture laboratory in a national stem cell research facility was chosen for study. This laboratory was situated in a building which had central airconditioning but the absence of a HEPA-enabled air handling unit resulted in frequent contamination of tissue culture samples. A handheld air sampler (SAS Super 100) was used, which could sample 100 liters of air per minute. Tryptic Soy Agar and Sabouraud dextrose agar plates were used to sample bacteria and fungi, respectively from the air. A fixed volume of air was sampled using the bio-sampler. Plates were placed in and removed from the bio-sampler in an aseptic manner. Plates were incubated at $25 \pm 2\,^{\circ }\hbox {C}$ (for fungal cultivation) and $37 \pm 2\,^{\circ }\hbox {C}$ (for bacterial cultivation) for 48 h. Post-incubation, the number of colonies appeared were enumerated and converted to CFU/m$^3$ using statistical conversion provided by the manufacturer. Control plates were used to ensure the sterility of the entire process.

## Supplementary Information

Supplementary Information.
