# Supplementary material for: Novel non intrusive continuous use ZeBox technology to trap and kill airborne microbes
Source: Sci Rep. 2021 Nov 23;11:22779. doi: 10.1038/s41598-021-02184-4 (PMC8610990; doi:10.1038/s41598-021-02184-4)
Supplement: Supplementary file 1 — Supplementary Information. [file 41598_2021_2184_MOESM1_ESM.pdf]

Novel non intrusive continuous use ZeBox technology to trap and kill airborne microbes

Kruttika S. Phadke[1,2], Deepak G. Madival[1,3], Janani Venkataraman[1], Debosmita Kundu[1], K. S. Ramanujan[1], Nisha Holla[1], Jaywant Arakeri[3], Gaurav Tomar[3], Santanu Datta[1,4], and Arindam Ghatak*[1]

[1] *Biomoneta Research Private Limited, Bangalore, India 560065*
[2] *Department of Veterinary Microbiology and Preventive Medicine, Iowa State University, Ames, IA, 50011, USA*
[3] *Mechanical engineering department, Indian Institute of Science, Bangalore, India 560012*
[4] *Bugworks Research, Bangalore, India 560066*

*Corresponding author: arindam@biomoneta.com

Supplementary information

S1. Range of microbes. To analyze microbe's motion in laminar flow between electrode-plates we adopt the following approximations: (1) The flow is identical to that between infinitely wide plates, which is justified because $W/H \gg 1$, where H is the gap between electrode-plates and W is their width (perpendicular to the flow direction); (2) The flow is fully developed, which is justified because $L/H \gg 1$, where L is the length of electrode-plates along the flow direction; (3) The microbes move with the flow except when electric force acts on them, which is justified because the Stokes number of microbes (a measure of its inertial response to changes in the flow) is $\ll 1$; (4) Self weight of microbes is negligible compared to the electric forces acting on them, because of their extremely small size ($< 5\ \mu$m).

The orientation of our coordinate system is shown in Fig. 1. A steady, unidirectional, incompressible, fully-developed flow is governed by [1]:

$$\frac{du}{dx} = 0 \qquad \text{(Mass conservation)} \tag{1}$$

$$\frac{dp}{dx} = \mu \frac{d^2u}{dz^2} \qquad \text{(Momentum conservation)} \tag{2}$$

where u is the flow velocity along X direction, p is pressure and μ is dynamic viscosity of the fluid. Since, subject to our assumptions, u depends only on z, mass conservation in supplementary equation (1) is automatically satisfied.

Since the flow is induced by imposing a pressure difference between the ends of the electrode-plates, the pressure gradient dp/dx is a constant. Therefore, momentum conservation in supplementary equation 2 is satisfied if u is a quadratic function of z. We assume it to be of the form, $u = Az(H - z)$, because this automatically satisfies the no-slip boundary condition on the electrode-plates located at $z = 0, H$. The constant A is determined by computing the volumetric flow rate and equating it to the known value Q, $W \int_0^H dz\ u = Q$, which yields:

$$u(z) = \frac{6Q}{WH^3}\ z(H - z) \tag{3}$$

where Q is the volumetric flow rate of air between the electrode-plates. The flow velocity varies only along Z direction, being maximum midway between the plates and zero at the plates themselves (no-slip condition). Because the Reynolds number of microbe's motion is $\ll 1$, due to its small size and small speeds, only Stokes drag force is exerted by the ambient fluid, $F_{\text{drag}} = 6\pi\mu wa$, where μ is the dynamic viscosity of air. We have assumed that the microbe can be approximated by an equivalent sphere of radius a. The drag counterbalances the electric force on the microbe, $F_{\text{electric}} = qE$, where q is the surface charge on the microbe and E is the strength of the applied electric field. Equating the two forces yields for the settling velocity:

$$w = \frac{qE}{6\pi\mu a} \tag{4}$$

While drifting towards the electrode-plate the microbe also travels a distance R in the flow direction, which we call its "range", refer Fig. 1. If z_0 is the initial distance of the microbe from the attracting electrode-plate at its entrance $x = 0$, then the time T needed for the microbe to hit the electrode-plate is, $T = z_0/w$. After time t, the vertical location of the microbe initially located at z_0 will be $z = z_0 - wt$. Then, from supplementary equation (3), the microbe's streamwise speed at that time will be $u(z_0 - wt)$. The microbe will hit the electrode-plate in time $T = z_0/w$ (assuming sufficient plate length). Therefore the range of the microbe beginning at location z_0 is given by $R(z_0) = \int_0^T dt\ u(z_0 - wt)$. Changing the integration variable to $z = z_0 - wt$ transforms the integral to: $R(z_0) = w^{-1} \int_0^{z_0} dz\ u(z)$. Substituting from supplementary equation (3) and integrating yields:

$$R(z_0) = \frac{6Qz_0^2}{WH^3w}\left(\frac{H}{2} - \frac{z_0}{3}\right), \qquad 0 \leq z_0 \leq H \tag{5}$$

The microbe that is farthest from the attracting electrode-plate, i.e. at $z_0 = H$, has the maximum range:

$$R_{\max} = \frac{Q}{Ww} \tag{6}$$

All the microbes entering ZeBox will hit the electrode-plate if its length is not less than the maximum range of the microbes, i.e. if $L \geq R_{\max}$. Supplementary

equation 5 for the range is visualized more easily if we divide it through by $R_{\max}$, supplementary equation 6, and rewrite it in the following dimensionless form:

$$\frac{R}{R_{\max}} = 6\left(\frac{z_0}{H}\right)^2\left(\frac{1}{2} - \frac{(z_0/H)}{3}\right), \qquad 0 \le \frac{z_0}{H} \le 1 \tag{7}$$

Assuming that all the trapped microbes are killed, supplementary equation 7 plotted in supplementary Fig. 1 completely determines the microbicidal efficiency of ZeBox, as per the present model. Here, "microbicidal efficiency" is defined as the fraction of microbes entering electrode-plates that hit it and are inactivated, assuming a uniform distribution at the entrance. Using supplementary figure 1, the microbicidal efficiency is found as follows. We first compute $L/R_{\max}$ given the operating parameters. If $L/R_{\max} \ge 1$, then the microbicidal efficiency is of course 100%. Otherwise, we locate its value on the vertical axis of supplementary figure 1 and using the curve find the corresponding value on the horizontal axis, which gives the microbicidal efficiency. Therefore, $L/R_{\max}$ alone determines the microbicidal efficiency of ZeBox according to the present model.

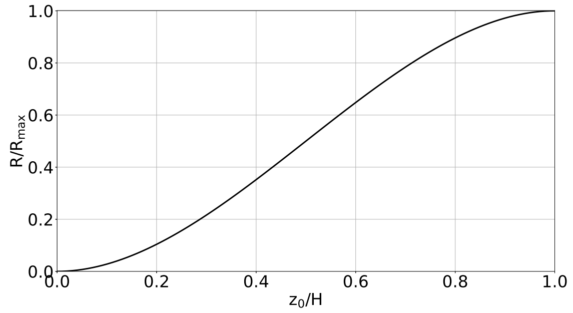

Supplementary figure 1: **Efficiency curve for ZeBox.** A microbe initially at distance z_0/H from the attracting electrode-plate hits it at a distance $R/R_{\max}$. (created using Matplotlib module in python language [2])

S2. Microbicidal efficiency of ZeBox. A microbe in an ionic solution is surrounded by a diffuse double layer of ions of molecular dimensions. The Debye length (κ^{-1}), which is a measure of the thickness of the double layer, lies in the range: $1 < \kappa^{-1} < 10$ nm [3]. Since the microbe's size $a \sim 1$ μm, $\kappa a \gg 1$ for microbes. The magnitude of the measured zeta potential (ζ) of microbes in phospate-buffer solution lies in the range 1 to 30 mV. Considering the worst-case-scenario, we may take $\zeta = 1$ mV and $\kappa a = 100$. The number of elementary charges n on the microbe may then be estimated as [3]:

$$n \approx \frac{4\pi\epsilon_r\epsilon_0\zeta a(1+\kappa a)}{e} \tag{8}$$

where ϵ_r is the dielectric constant of the solution, $\epsilon_0 = 8.9 \times 10^{-12}$ F/m is the permittivity of vaccuum and $e = 1.6 \times 10^{-19}$C is the magnitude of the

elementary charge. Supplementary equation (8) is derived from the linearized Poisson-Boltzmann equation governing the variation of electric potential due to distribution of ions in the diffuse layer surrounding a charged sphere; the linearization is a consequence of the Debye-Hückel approximation which is applicable when zeta potential is small (compared to $\sim$ 25 mV at 25 ^{0}C) [3]. For a measured dielectric constant of 78.5 for the buffer solution, supplementary equation (8) reveals $n > 5000$ elementary charges. Even allowing for an order-of-magnitude error and assuming $n > 500$ instead, supplementary equation (4) yields a settling velocity of $w > 7$ cm/s, for $E = 3$ kV/cm in air.

Since the flow rate between a pair of electrode-plates is $Q < 3$ cfm and the electrode-plate width $W = 10.9$ cm, supplementary equation (6) shows that $R_{\max} < 19$ cm. In comparison, ZeBox employs 30 cm long electrode-plates. Although in theory it implies 100 % microbicidal efficiency for ZeBox, the present model is only approximate because it does not account for the effects of possible turbulence in the flow and slippage of microbes on the surface. In reality, as mentioned in the Results section, we obtain 83-99 % microbicidal efficiency for ZeBox as deduced from the measured microbial load reduction.

References

[1] Kundu P.K., Cohen I.M., Hu H.H. Fluid Mechanics. Academic press, London (pp. 516); 2008.

[2] Hunter JD. Matplotlib: A 2D graphics environment. Computing in science & engineering. 2007 May 1;9(03):90-5.

[3] Hiemenz P. C., Rajagopalan R. Principles of Colloid and Surface Chemistry.
